# The impact of digital literacy on health behaviors among middle-aged and older adults: the mediating roles of proactive health awareness and social capital

**DOI:** 10.3389/fpubh.2026.1735211

**Published:** 2026-02-12

**Authors:** Xue Tang, Huiyan Peng, Jie Li, Mingshu Si

**Affiliations:** 1 School of Public Administration and Sociology, Jiangsu Normal University, Xuzhou, China; 2 Department of Social Medicine and Health Management, School of Public Health, Cheeloo College of Medicine, Shandong University, Jinan, China; 3 NHC Key Lab of Health Economics and Policy Research (Shandong University), Jinan, China; 4 Center for Health Management and Policy Research, Shandong University (Shandong Provincial Key New Think Tank), Jinan, China; 5 School of Health Management, Fujian Medical University, Fuzhou, China; 6 Health and Elderly Care Professional Committee of Fujian Research Association for Medical and Health System, Fuzhou, China

**Keywords:** digital literacy, health behavior, middle-aged and older adults, proactive health awareness, social capital

## Abstract

**Background:**

With the rapid acceleration of population aging in China, promoting healthy behaviors among middle-aged and older adults has become an urgent public health priority. Digital literacy, as a key skill in the digital era, may influence health behaviors directly or indirectly through psychological and social pathways. This study analyzes the impact of digital literacy on health behaviors among middle-aged and older adults, focusing on the mediating roles of proactive health awareness and social capital.

**Methods:**

Using data from the 2021 Chinese General Social Survey (CGSS), this study constructed a composite digital literacy index based on the entropy weight method. A total of 1,458 respondents aged 45 and above were included. Health behaviors were measured by dietary and exercise indicators, while proactive health awareness and social capital were modeled as mediators. Ordinary least squares regression, ordinal logistic regression, and ordered probit regression were applied to analyse the direct and indirect effects of digital literacy on health behaviors.

**Results:**

Digital literacy had no significant direct effect on health behaviors (*β* = 0.098, *p* > 0.05). Mediation analysis indicated significant indirect effects: higher digital literacy was associated with greater proactive health awareness (indirect effect = 0.085, *p* < 0.01) and with greater social capital (indirect effect = 0.049, *p* < 0.05), both of which were linked to healthier behaviors. A sequential pathway (digital literacy → proactive health awareness → social capital → health behavior) was also significant (indirect effect = 0.011, *p* < 0.01). Age-stratified analysis showed that these mediating effects were significant only among middle-aged adults (45–59 years) and not among older adults (≥60 years).

**Conclusion:**

Among middle-aged and older Chinese adults, digital literacy appears to influence health behaviors mainly by enhancing health awareness and expanding social support. These findings imply that interventions to promote healthy aging should combine digital skills training with health education and community support networks. The observed age differences suggest that such strategies may need to be tailored to different age groups.

## 1 Introduction

With the rapid acceleration of population aging in China, health challenges among older adults have become increasingly prominent. According to the 2024 National Bulletin on the Development of Undertakings for the Aged released by the Ministry of Civil Affairs and the National Working Commission on Aging, by the end of 2024, the number of people aged 60 and above in China had reached 310 million, accounting for 22.00% of the total population (1). This indicates that China has already entered a moderately aged society. As people grow older, they are more likely to face chronic diseases, functional decline, and health inequalities (2). These issues not only severely affect their quality of life but also pose significant challenges to the sustainability of the medical security system and the broader socioeconomic development (3). Enhancing the health status of older adults is therefore not only vital to individual and family well-being but also an essential guarantee for improving overall social welfare. Achieving “healthy aging” has become a strategic priority for China in addressing population aging and promoting high-quality demographic development.

Health challenges associated with population aging are not exclusively confined to individuals aged 60 and above. Substantial epidemiological evidence indicates that the middle-aged population is at a critical transitional stage of the life health course. During this stage, chronic conditions begin to emerge (4, 5), and lifestyle patterns gradually become fixed. The health behaviors and habits formed at this critical period have been shown to significantly predict individuals’ later-life health status, the onset of chronic diseases, and the degree of subsequent disability (6), thereby exerting long-term impact on overall well-being. Therefore, the inclusion of the middle-aged population in this study aims to implement a ‘front-loading’ health intervention strategy to prevent or delay the progression of functional decline and chronic diseases. Compared with treating diseases already established in old age, this preventive approach is considered more cost-effective in the field of public health.

To address the health challenges brought about by population aging, the Chinese government introduced the “Healthy China 2030” strategy, which explicitly emphasizes improving health behaviors and enhancing health literacy as key strategies to achieve the goal of universal health (7). Against this policy backdrop, effectively promoting healthy behaviors among middle-aged and older adults has become a central issue in implementing the “Healthy China 2030” strategy. Health behavior is a critical determinant of health maintenance and promotion, encompassing aspects such as balanced diet, regular physical activity, and access to and utilization of health information. Previous studies have demonstrated that positive health behaviors significantly reduce the risk of chronic diseases and improve the physical and mental health of older adults (8–10). However, in practice, Chinese older adults continue to face shortcomings in this regard: participation in physical exercise remains relatively low, dietary structures are often sub-optimal, and their ability to acquire and use health information is limited (11, 12). These problems are closely associated with factors such as lower levels of education, weaker digital skills, and limited social support networks among older adults (13, 14). Therefore, fostering healthy behaviors in the older population has become an urgent public health and social policy priority.

In the context of rapid digitization and informatization, digital technologies play an increasingly important role in daily life and health management. In recent years, China has vigorously promoted the construction of a “Digital China” and the development of “Internet Plus Healthcare,” aiming to leverage digital tools to enhance accessibility and equity in health services. Nevertheless, older adults generally face a pronounced “digital divide,” with limited digital literacy skills (15–17). Digital literacy not only determines whether they can access the internet and use smart devices, but also directly affects their ability to acquire, evaluate, and apply health-related information. Empirical evidence has shown that individuals with higher levels of digital literacy are more likely to proactively obtain health knowledge, seek medical consultation via online platforms, and adopt healthier lifestyles in their daily routines (18–20). Thus, digital literacy may represent a crucial factor in promoting behavioral changes toward healthier living among middle-aged and older population.

Current research on digital literacy has primarily focused on younger or highly educated groups, yielding substantial findings in areas such as information acquisition, mechanisms of improvement, and career development (21, 22). Meanwhile, some scholars have begun to examine the “digital divide” among older adults, exploring its effects on life satisfaction, social participation, and cognitive function (23, 24). However, in China, empirical studies on the impact of digital literacy on the older adults remain insufficient, particularly those employing multidimensional measures of digital literacy. Existing research has mainly addressed the direct effects of digital literacy on health (25, 26), with relatively limited attention to the underlying mechanisms. In other words, there is a lack of systematic empirical analysis of how digital literacy indirectly influences health management behaviors of older adults through psychological and social pathways. In particular, within the Chinese context, proactive health awareness and social capital may play crucial mediating roles, yet relevant investigations remain scarce. Therefore, an in-depth exploration of the mechanisms through which digital literacy influences health management behaviors among middle-aged and older adults will not only enrich theoretical understanding but also provide evidence-based insights for improving public policy and practice.

Building on the above background and research gap, this study aims to examine the impact of digital literacy on health management behaviors among middle-aged and older population, as well as its underlying mechanisms. Specifically, using survey data, a composite digital literacy index is constructed to analyze, first, the direct effects of digital literacy on health behaviors. Second, by introducing proactive health awareness and social capital as mediating variables, the study investigates how digital literacy indirectly influences health behaviors through psychological cognition and social network pathways, thereby revealing its internal mechanisms. In terms of significance, this research contributes to advancing theoretical knowledge of the relationship between digital literacy and health management behaviors, enriching scholarly understanding in the field of digital health under the context of an aging society. Moreover, it provides empirical evidence for the formulation of public policies, promoting inclusive applications of digital technology among older adults and contributing to the achievement of the “Healthy China 2030” strategic goals.

### 1.1 Theoretical analysis and research hypotheses

In the context of the deep integration of digital transformation and population aging, digital literacy is defined as the comprehensive ability of individuals to effectively obtain, understand, evaluate, and utilize digital information and tools to achieve their goals. It encompasses dimensions such as access ability, information retrieval ability, application ability, and information judgment ability (27). For middle-aged and older adults, digital literacy not only determines whether they can smoothly enter the digital society but also directly affects their ability and effectiveness in obtaining health information, utilizing health services (such as online consultation, telemedicine, and health data tracking), and using health management tools (such as health apps and wearable devices), thereby potentially shaping their health-related behaviors and lifestyle choices (28, 29).

**Figure 1:**
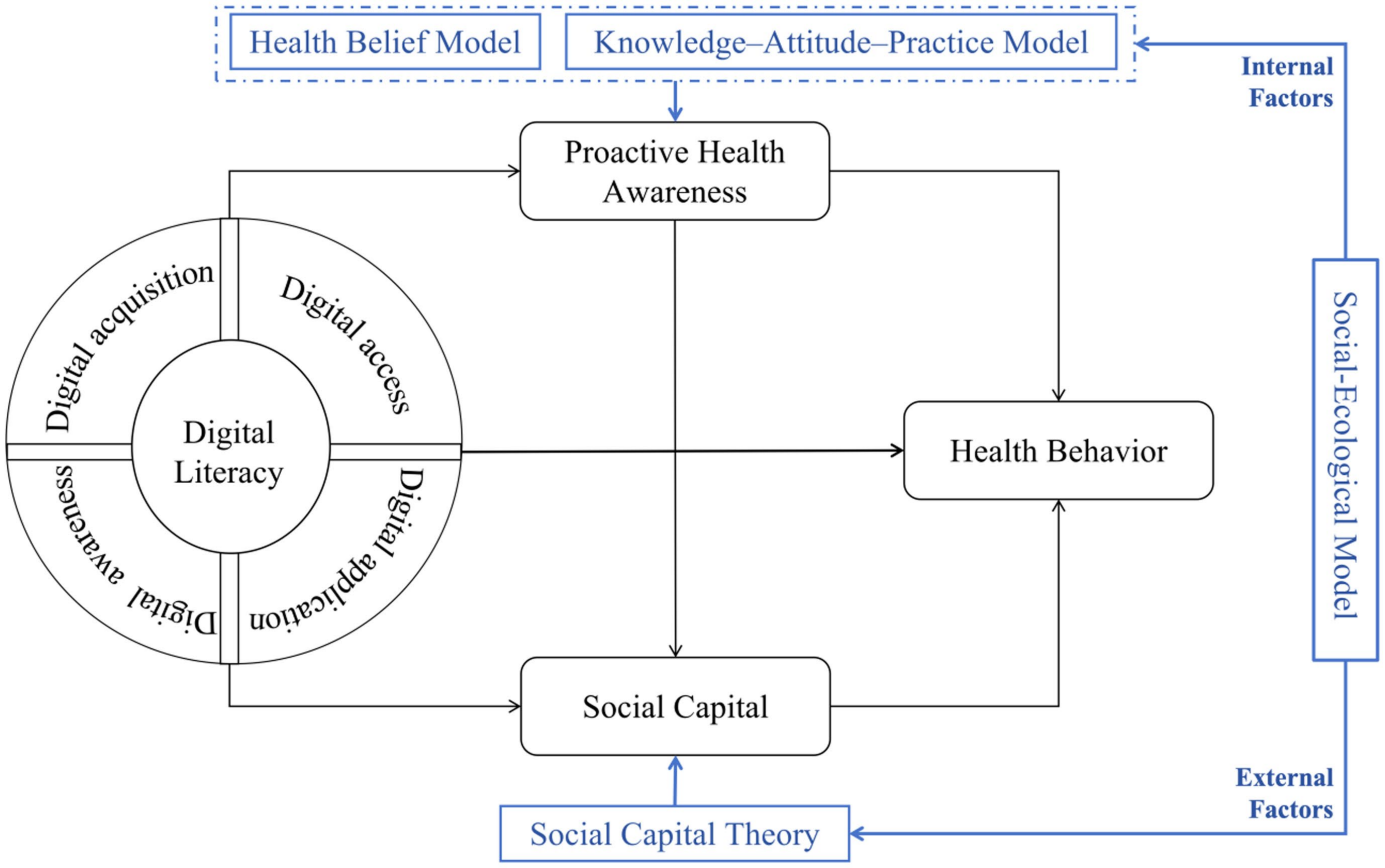
Theoretical analysis diagram.

To examine the complexity of how digital literacy (DL) influences the health behaviors of middle-aged and older adults, our study integrates the Social-Ecological Model (SEM), the Knowledge–Attitude–Practice (KAP) Model, the Health Belief Model (HBM), and Social Capital Theory to develop a comprehensive framework of “digital literacy—psychological mechanism—social mechanism—health behavior” (see Figure 1). This integration is based on the recognition that the determinants of health behavior are multi-level and multi-dimensional, and single theory cannot fully capture this complexity in the digital era (30). First, the SEM serves as the overarching conceptual framework and provides theoretical coherence. SEM proposes that health behavior results from the interaction of factors at multiple levels, including individual, interpersonal, community, and societal levels. It supports the need to examine both internal cognitive factors (explained by HBM and KAP model) and external social environmental factors (explained by Social Capital Theory), thereby forming the theoretical foundation for the dual-mediation model in this study. Second, we integrate the KAP model and HBM to build and explain the individual cognitive–psychological mechanism. The KAP model emphasizes the progression from knowledge to attitudes and then to practices, while HBM focuses on individuals’ beliefs and perceptions about disease. As a key capability for information acquisition, digital literacy directly shapes an individual’s health knowledge, beliefs, and perceptions. The combination of these psychological elements forms the core mediating variable in this study—proactive health awareness. This integration clarifies how digital skills translate into internal motivation for health behavior. Finally, Social Capital Theory is used to explain the social-relational mechanism. Social Capital Theory focuses on external support and resources. It explains how higher levels of digital literacy enable middle-aged and older adults to more effectively build and maintain digital social connections, allowing them to obtain health-related support and resources. This theoretical perspective aligns with SEM’s emphasis on interpersonal and environmental influences and addresses the limitations of cognitive models in explaining external support mechanisms.

*H1*: Digital literacy level has a significant positive impact on the health management behaviors of middle-aged and older adults.

This hypothesis is based on the the resource-accessibility perspective of the Social-Ecological Model (SEM) and individual-level assumptions of the KAP model. Theoretically, digital literacy (DL) functions as a crucial resource (SEM) and a prerequisite for Practice (KAP model). Individuals with higher DL can more conveniently access health information and utilize complex digital health services (such as online consultation, telemedicine, and health data tracking) (27). This ability directly reduces information asymmetry and behavioral accessibility barriers to services, such as prescription renewal or health monitoring, thereby directly facilitating the adoption of positive health behaviors.

*H2*: Digital literacy indirectly promotes health behaviors by enhancing proactive health awareness.

This hypothesis is rooted in the HBM and KAP model, emphasizing the information-cognition-behavior transmission path. Theoretically, digital literacy (DL) is a prerequisite for information access. Higher DL empowers individuals to effectively obtain and evaluate health information, thereby establishing the “Knowledge” base in the KAP Model. Concurrently, DL enhances perceived susceptibility, perceived benefits, and self-efficacy (core elements of HBM). The convergence of these psychological elements constitutes Proactive Health Awareness, which, in turn, drives health practice (31). Previous empirical studies have shown that improvements in digital health literacy significantly increase proactive health information–seeking behavior (32), and this active digital engagement and enhanced health self-efficacy are positively associated with better health management behaviors among middle-aged and older adults (33).

*H3*: Digital literacy indirectly promotes health behaviors by enhancing social capital.

This hypothesis is based on social capital theory. Unlike the HBM and KAP model, which focus on internal cognitive drivers, social capital theory emphasizes external support. Higher digital literacy allows middle-aged and older adults to build and maintain digital social connections, which enables them to acquire and mobilize social capital, including reciprocity, trust, and support. These external resources play an important role in the adoption and maintenance of health behaviors by providing health norms, behavioral role models, and emotional support (28, 30). Empirical evidences support that higher levels of digital engagement are associated with greater social support, and increases in social capital are significantly linked to higher participation in physical activity and healthier dietary habits among middle-aged and older adults (34–36).

*H4*: Proactive health awareness and social capital play a chain-mediated role between digital literacy and health behaviors.

This hypothesis proposes a sequential mediation that integrates both psychological and social mechanisms within the Social-Ecological Model (SEM) framework: Digital Literacy → Proactive Health Awareness → Social Capital → Health Behavior. Theoretically, digital literacy first enhances proactive health awareness by improving individuals’ ability to acquire, evaluate, and internalize health information, establishing the psychological mechanism. Crucially, individuals with stronger internal health awareness are motivated to actively seek external support. This motivation drives them to engage in health-related communication, participate in community or online health groups, and form supportive social networks, thereby increasing their social capital (the social mechanism). This accumulated social capital then provides essential informational and emotional support that ultimately facilitates the adoption and maintenance of health behaviors (30). This theoretical sequence emphasizes that cognitive activation precedes and drives social engagement in the pathway from digital capacity to health outcomes.

## 2 Materials and methods

### 2.1 Data source

The data utilized in this study were derived from the 2021 wave of the Chinese General Social Survey (CGSS), conducted by the China Survey and Data Center at Renmin University of China. As the first nationwide, comprehensive, and continuous academic survey project in China, the CGSS has been implemented since 2003, covering 31 provinces, autonomous regions, and municipalities across mainland China. It employs a rigorous stratified, multi-stage probability sampling method to collect data from over ten thousand households nationwide. By systematically and regularly gathering information on multiple aspects of Chinese society and individuals, the CGSS provides high-quality data to support research on social transformation in China and other topics of significant theoretical and practical importance.

The CGSS 2021 included a total of 8,148 valid samples. For the purposes of this study, the data were cleaned and filtered as follows: first, given that the target population was middle-aged and older adults, only respondents aged 45 years and above were retained; second, cases with missing values or extreme values on key variables were excluded. The final analytical sample comprised 1,458 respondents, which served as the basis for examining the impact of digital literacy on health management behaviors among middle-aged and older adults in China.

### 2.2 Measures

#### 2.2.1 Dependent variable: health behavior

The dependent variable is health behavior of middle-aged and older adults in China. Two questions were selected as proxy indicators of health behavior: “Do you often engage in at least 20 minutes of physical exercise that makes you sweat or breathe faster?” and “Do you often eat fresh fruits or vegetables?”. The response options were coded as follows: “Never” = 1, “Once a month or less” = 2, “Several times a month” = 3, “Several times a week” = 4, and “Every day” = 5. The scores from these two dimensions were then summed to generate a composite health behavior score for middle-aged and older adults.

#### 2.2.2 Independent variable: digital literacy

Digital literacy. This study conceptualized digital literacy across four dimensions: digital acquisition literacy, digital access literacy, digital application literacy, and digital awareness literacy, and measured each dimension using one corresponding survey item. Specifically, “Do you have a mobile phone for your personal use?” was used to measure digital acquisition literacy; “Have you accessed the internet in the past 6 months, including through a computer, mobile phone, or smart wearable device?” was used to measure digital access literacy; “In the past year, how often did you use the internet, including mobile internet access?” was used to measure digital application literacy; and “Is the internet, including mobile internet, your primary source of information?” was used to measure digital awareness literacy. Given that digital literacy is a multidimensional and complex construct, simply summing the items may not fully capture the differences and relative importance among indicators. Therefore, this study employed the entropy weight method to construct a composite digital literacy index. The entropy weight method objectively assigns weights based on the characteristics of the data and accounts for interactions between indicators, thus producing a more scientifically and reasonably weighted composite index (37, 38). The selected indicators and corresponding items are presented in Table 1.

**Table 1:** Digital literacy.

Dimension	Question	Response coding
Digital acquisition literacy	Do you have a mobile phone for your personal use?	Yes = 1, no = 0
Digital access literacy	Have you accessed the internet in the past 6 months, including through a computer, mobile phone, or smart wearable device?	Yes = 1, no = 0
Digital application literacy	In the past year, how often did you use the internet (including mobile internet access)?	Never = 1, rarely = 2, sometimes = 3, often = 4, very frequently = 5
Digital awareness literacy	Is the Internet (including mobile Internet access) your main source of information?	Yes = 1, no = 0

#### 2.2.3 Mediating variables: proactive health awareness and social capital

##### 2.2.3.1 Proactive health awareness

Proactive health awareness was measured using two survey items. The first item asked: *“In the past 12 months, how frequently have you searched online for health or medical information for yourself or others?”* Response options included: 1 = never, 2 = hardly ever, 3 = a few times a year, 4 = a few times a month, 5 = a few times a week, 6 = once a day, 7 = several times a day. The second item asked: *“In the past 12 months, how often have you searched online for information on healthy lifestyles?”* Response options ranged from 1 = never, 2 = rarely, 3 = sometimes, 4 = often, to 5 = very frequently. An entropy weighting method was employed to integrate these two indicators into a composite index of proactive health awareness.

##### 2.2.3.2 Social capital

Social capital was measured using two questions. The first asked: *“How often do you engage in social or recreational activities (e.g., visiting each other’s homes, watching TV, dining, playing cards, etc.) with your neighbors?”* The second asked: *“How often do you engage in such activities with other friends?”* Response categories were coded as: 1 = never, 2 = once a year or less, 3 = a few times a year, 4 = about once a month, 5 = a few times a month, 6 = 1–2 times a week, 7 = almost every day. The scores from these two items were summed to obtain a composite measure of social capital among middle-aged and older adults.

#### 2.2.4 Covariates

To reduce potential estimation bias arising from missing dependent variables, several covariates were included in the model. These variables were derived from the survey data on individual characteristics of middle-aged and older adults, including gender, age, ethnicity, educational, Hukou, marital status, individual annual income in the last year, and self-rated health status.

The meaning, assignment, and descriptive statistics of the variables used in this study are shown in Table 2.

**Table 2:** Descriptive statistics of variables.

Variable	Description	Mean	S.D.^1^
Dependent variable			
Engaged in physical exercise	Never = 1, once a month or less = 2, several times a month = 3, several times a week = 4, every day = 5	2.84	1.71
Engaged in healthy diet	Never = 1, once a month or less = 2, several times a month = 3, several times a week = 4, every day = 5	4.59	0.82
Health behavior	Addition of the two above indicators	7.43	2.01
Independent variable			
Digital literacy	Comprehensive indicator	0.49	0.42
Mediating variables			
Proactive health awareness	Comprehensive indicator	0.19	0.25
Engage in social or leisure activities with neighbors	Never = 1, once a year or less = 2, several times a year = 3, once a month = 4, several times a month = 5, once or twice a week = 6, every day = 7	3.83	2.32
Engage in social or leisure activities with friends	Never = 1, once a year or less = 2, several times a year = 3, once a month = 4, several times a month = 5, once or twice a week = 6, every day = 7	3.44	2.03
Social capital	Addition of the two above indicators	7.27	3.77
Covariates			
Gender	Male = 0, female = 1	0.55	0.50
Age (years)	45–92 years old	61.52	10.21
Ethnicity	Hans = 0, minority = 1	0.06	0.24
Education	Illiteracy = 1, primary school = 2, junior school = 3, high school and above = 4	2.69	1.01
Hukou	Agriculture = 0, non-agriculture = 1	0.40	0.49
Marital status	Single = 0, married = 1	0.80	0.40
Individual annual income (yuan)	Less than 10,000 = 1, 10,000–29,999 = 2, 30,000 and above = 3	2.00	0.88
Health status	Very poor = 1, poor = 2, fair = 3, healthy = 4, very healthy = 5	3.24	1.08

1 S.D. stands for standard deviation.

### 2.3 Model setting

To analyze the impact of digital literacy on the health behavior of middle-aged and older adults in China, this study first establishes a baseline regression model. The primary model is estimated using Ordinary Least Squares (OLS), specified as follows:

$HB_{i}=\beta_{0}+\beta_{1}DL_{i}+\beta_{n}\text{Contro}l_{i}+\varepsilon_{i}$ (1)

where HB_i_ represents the health behavior score of individual i; β_0_ is the intercept; DL_i_ denotes the digital literacy level of individual i; Control_i_ represents a vector of covariates, including gender, age, ethnicity, educational, Hukou, marital status, individual annual income, and health status; and ε_i_ is the random disturbance term. The key parameter of interest, *β*_1_, captures the effect of digital literacy on health behavior.

To further explore the underlying mechanisms, a mediation effect model is introduced. The mediating variables include proactive health awareness and social capital of middle-aged and older adults. The regression equations are specified as follows:

$M_{i}=\alpha_{0}+\alpha_{1}DL_{i}+\alpha_{n}\text{Contro}l_{i}+\mu_{i}$ 2)

$Y_{i}=\gamma_{0}+\gamma_{1}DL_{i}+\gamma_{2}M_{i}+\gamma_{n}\text{Contro}l_{i}+\theta_{i}$ (3)

In Equations (2) and (3), *M_i_* denotes the mediating variables of individual *i*, including proactive health awareness and social capital; *α*_0_ and *γ*_0_ are constants; *α*_1_ measures the effect of digital literacy on the mediators; *γ*_2_ captures the effect of the mediators on health behavior; and *γ*_1_ represents the direct effect of digital literacy on health behavior after controlling for the mediators. *μ_i_* and *θ_i_* are the random disturbance terms.

## 3 Results

### 3.1 Correlation analysis of key variables

Table 3 presents the Pearson correlation coefficients among the key study variables. Health behavior was positively and significantly correlated with digital literacy (*r* = 0.125, *p* < 0.001), proactive health awareness (*r* = 0.173, *p* < 0.001) and social capital (*r* = 0.127, *p* < 0.001). Digital literacy was also positively associated with proactive health awareness (*r* = 0.592, *p* < 0.001), and social capital (*r* = 0.119, *p* < 0.001). Additionally, proactive health awareness and social capital were positively correlated (*r* = 0.126, *p* < 0.001). These results indicate that all variables were positively interrelated, providing a statistical foundation for subsequent mediation analyses examining the roles of proactive health awareness and social capital in the relationship between digital literacy and health behavior.

**Table 3:** Pearson correlation coefficients between key study variables (*N* = 1,458).

Variables	Health behavior	Digital literacy	Proactive health awareness	Social capital
Health behavior	1.000			
Digital literacy	0.125^***^	1.000		
Proactive health awareness	0.173^***^	0.592^***^	1.000	
Social capital	0.127^***^	0.119^***^	0.126^***^	1.000

***p < 0.001, **p < 0.01, *p < 0.05.

### 3.2 Impact of digital literacy on the health behavior of middle-aged and older adults

Models 1 and 2 in Table 4 examined the effect of digital literacy on proactive health awareness. The unadjusted model (Model 1) showed that digital literacy had a significant positive effect on proactive health awareness (*β* = 0.354, *p* < 0.001). After controlling for covariates including gender, age, ethnicity, education level, Hukou, marital status, individual annual income, and health status (Model 2), the positive effect of digital literacy on proactive health awareness remained significant (*β* = 0.296, *p* < 0.001), indicating that individuals with higher levels of digital literacy tend to have stronger proactive health awareness.

**Table 4:** Impact of digital literacy on the health behavior of middle-aged and older adults.

Variables	Proactive health awareness		Social capital		Health behavior	
	Model 1 (unadjusted)	Model 2 (adjusted)	Model 3 (unadjusted)	Model 4 (adjusted)	Model 5 (unadjusted)	Model 6 (adjusted)
Digital literacy	0.354*** (0.012)	0.296*** (0.016)	0.619* (0.291)	0.772* (0.333)	0.133 (0.157)	0.073 (0.174)
Proactive health awareness	—	—	1.291** (0.481)	1.445** (0.495)	1.155*** (0.250)	0.777** (0.261)
Social capital	—	—	—	—	0.056*** (0.014)	0.056*** (0.014)
Gender (male^Ref^)						
Female		0.008 (0.011)		0.012 (0.207)		0.028 (0.108)
Age/years		0.001 (0.001)		−1.086** (0.370)		0.007 (0.006)
Ethnicity (Hans^Ref^)						
Minority		0.009 (0.018)		0.013 (0.326)		0.050 (0.204)
Education (illiteracy ^Ref^)						
Primary school		0.018 (0.012)		0.104 (0.339)		0.050 (0.204)
Junior school		0.057*** (0.014)		−0.266 (0.388)		0.050 (0.204)
High school and above		0.105*** (0.018)		−0.578* (0.256)		0.050 (0.204)
Hukou (agriculture^Ref^)						
Non-agriculture		0.031* (0.014)		−0.165 (0.263)		0.247 (0.128)
Marital status (single^Ref^)						
Married		0.012 (0.012)		−0.028 (0.284)		0.277* (0.133)
Individual annual income/yuan (less than 10,000^Ref^)						
10,000–29,999		−0.004 (0.013)		0.261 (0.286)		−0.008 (0.145)
30,000 and above		0.027 (0.015)		0.663 (0.455)		−0.107 (0.152)
Health status (very poor^Ref^)						
Poor		−0.003 (0.018)		0.863* (0.431)		0.441 (0.252)
Fair		0.012 (0.018)		0.927* (0.433)		0.879*** (0.239)
Healthy		0.022 (0.019)		1.028* (0.495)		0.876*** (0.241)
Very healthy		0.024 (0.023)		1,458 0.034		1.029*** (0.269)
Observations	1,458	1,458	1,458	0.772* (0.333)	1,458	1,458
R-squared	0.350	0.395	0.019	1.445**	0.042	0.078

Robust standard errors in parentheses; ***p < 0.001, **p < 0.01, *p < 0.05; All variance inflation factors (VIF) were below 5, indicating no serious multicollinearity.

Models 3 and 4 in Table 4 examined the effect of digital literacy on social capital. In the unadjusted model (Model 3), digital literacy was significantly positively associated with social capital (*β* = 0.619, *p* < 0.05). This positive association further strengthened after controlling for covariates (*β* = 0.772, *p* < 0.05), suggesting that older adults with higher digital literacy generally possess greater social capital.

Models 5 and 6 in Table 4 analyzed the effects of digital literacy on health behavior. In the unadjusted model (Model 5), both proactive health awareness (*β* = 1.155, *p* < 0.001) and social capital (*β* = 0.056, *p* < 0.001) significantly promoted health behavior, whereas the direct effect of digital literacy was not significant (*β* = 0.133, *p* > 0.05). After controlling for covariates (Model 6), proactive health awareness (*β* = 0.777, *p* < 0.01) and social capital (*β* = 0.056, *p* < 0.001) remained significant predictors of health behavior, while the direct effect of digital literacy remained non-significant (*β* = 0.073, *p* > 0.05), indicating that proactive health awareness and social capital may mediate the relationship between digital literacy and health behavior.

Overall, the findings suggest that digital literacy does not have a significant direct effect on health behavior, but it may indirectly promote health behavior among older adults through enhancing proactive health awareness and social capital, providing a solid statistical foundation for subsequent mediation analyses.

### 3.3 Robustness analysis

In the robustness analysis (Supplementary Table S1), ordinal logistic regression (OLR) was first employed to examine the effects of digital literacy, proactive health awareness, and social capital on health behavior. In the unadjusted model (Model 7), proactive health awareness (*β* = 1.025, *p* < 0.001) and social capital (*β* = 0.051, *p* < 0.001) showed significant positive effects on health behavior, whereas the direct effect of digital literacy was not statistically significant (*β* = 0.134, *p* > 0.05). After including covariates (Model 8), proactive health awareness (*β* = 0.688, *p* < 0.01) and social capital (*β* = 0.053, *p* < 0.001) remained significantly associated with better health behavior, while digital literacy continued to exert no significant direct effect (*β* = 0.099, *p* > 0.05).

Given that the dependent variable—health behavior—is an ordered categorical variable, ordered probit regression (Oprobit) was further applied for robustness checks. In the unadjusted model (Model 9), proactive health awareness (*β* = 0.634, *p* < 0.001) and social capital (*β* = 0.031, *p* < 0.001) again demonstrated significant positive associations with health behavior, whereas digital literacy remained non-significant (*β* = 0.054, *p* > 0.05). After the inclusion of covariates (Model 10), proactive health awareness (*β* = 0.439, *p* < 0.01) and social capital (*β* = 0.031, *p* < 0.001) continued to exhibit robust positive effects, while the direct effect of digital literacy was still not statistically significant (*β* = 0.027, *p* > 0.05).

Overall, the results from both OLR and Oprobit models are highly consistent, indicating that proactive health awareness and social capital play crucial roles in promoting health behavior, whereas the direct effect of digital literacy on health behavior is not significant. These findings further confirm the robustness of the main regression results and provide additional support for the hypothesis that digital literacy may influence health behavior indirectly through mediating mechanisms such as proactive health awareness and social capital.

### 3.4 Dimensional analysis

Furthermore, since the dependent variables were replaced with each specific health behavior and given that these health behaviors are ordinal categorical variables, the analysis employed an ordinal logistic regression model. Supplementary Table S2 presents the regression results of digital literacy on the two types of health behaviors.

For physical exercise, the results from Model 11 (unadjusted) and Model 12 (adjusted) both indicate that the direct effect of digital literacy was not statistically significant, whereas proactive health awareness and social capital demonstrated stable positive effects. Specifically, proactive health awareness had a significant impact on physical exercise in Model 11 (*β* = 0.877, *p* < 0.001) and remained significant after controlling for demographic and socioeconomic variables in Model 12 (*β* = 0.616, *p* < 0.01), suggesting that a higher level of health awareness is an important factor in promoting participation in physical exercise. At the same time, social capital exhibited consistently significant positive effects in both Model 11 and Model 12 (*β* = 0.043, *p* < 0.001; *β* = 0.043, *p* < 0.001), indicating that individuals with higher levels of social capital are more likely to engage in physical exercise.

For healthy diet, the results from Model 13 (unadjusted) and Model 14 (adjusted) similarly show that the direct effect of digital literacy was not significant. Proactive health awareness had a positive impact in Model 13 (*β* = 0.278, *p* < 0.01), but this effect weakened and lost statistical significance in Model 14 after the inclusion of covariates, implying that its influence on dietary behavior may be constrained by other factors. In contrast, social capital maintained significant positive effects across both Model 13 and Model 14 (*β* = 0.013, *p* < 0.05; *β* = 0.013, *p* < 0.05), suggesting that social capital plays a sustained role in fostering healthy dietary practices.

### 3.5 Mediating effect analysis

**Figure 2:**
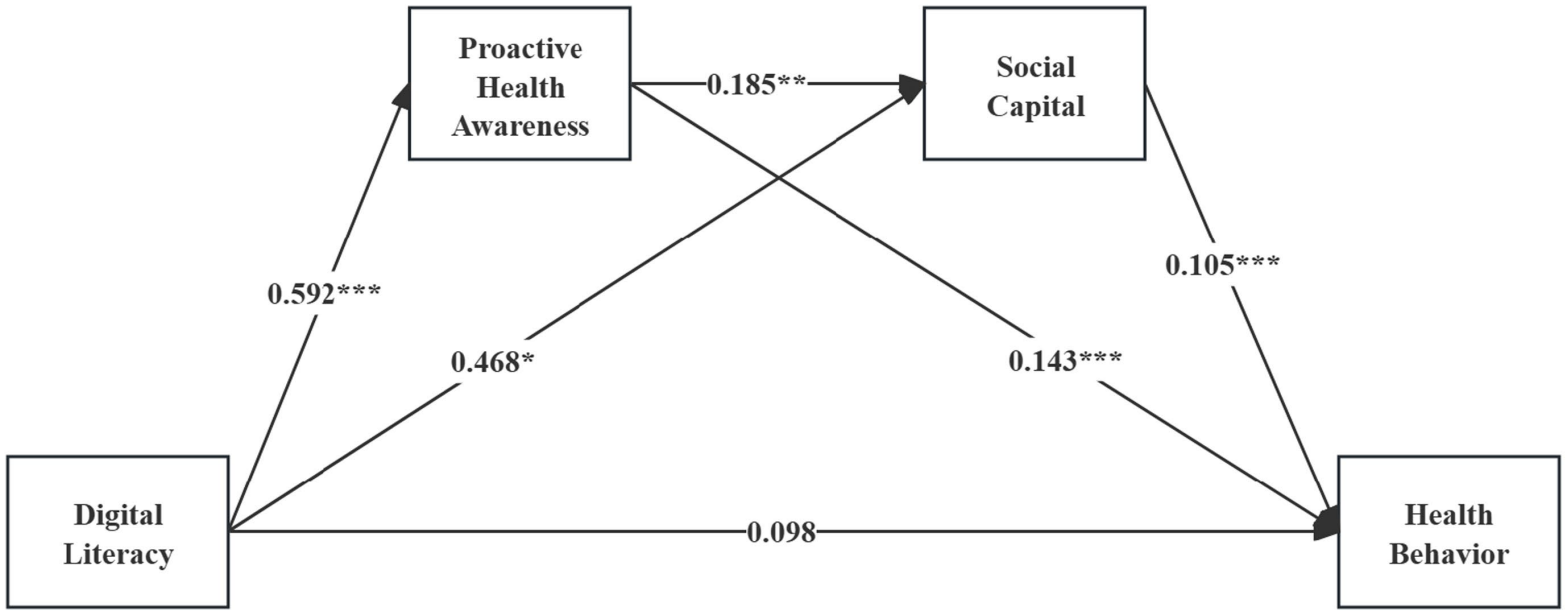
A multiple mediation model of the association between digital literacy and health behavior throng proactive health awareness and social capital. Standard path coefficients are shown, ***p < 0.001, **p < 0.01, *p < 0.05.

Figure 2 presents the multiple mediation model linking digital literacy to health behavior through proactive health awareness and social capital. The findings show that the total effect of digital literacy on health behavior is 0.243. The direct effect is 0.098, accounting for 40.33% of the total effect, and is not statistically significant (*p* > 0.05), indicating that digital literacy does not exert a direct predictive effect on health behavior.

In contrast, the overall indirect effect is significant (indirect effect = 0.145, representing 59.67% of the total effect), suggesting that digital literacy primarily influences health behavior through mediation pathways. Specifically: (1) the pathway Digital Literacy → Proactive Health Awareness → Health Behavior yields an indirect effect of 0.085, which constitutes 34.98% of the total effect (*p* < 0.01), identifying proactive health awareness as the central mediator; (2) the pathway Digital Literacy → Social Capital → Health Behavior produces an indirect effect of 0.049, representing 20.16% of the total effect (*p* < 0.05), suggesting a supplementary mediating role of social capital; (3) notably, the chained mediation pathway Digital Literacy → Proactive Health Awareness → Social Capital → Health Behavior has an indirect effect of 0.011, accounting for 4.53% of the total effect and reaching statistical significance (*p* < 0.01). This finding highlights that digital literacy fosters proactive health awareness, which in turn enhances social capital, thereby indirectly promoting health behavior through a sequential mechanism.

Taken together, the results indicate that the impact of digital literacy on health behavior operates predominantly through indirect mechanisms. Proactive health awareness not only plays a critical mediating role on its own but also facilitates the formation of social capital, thereby establishing a chained mediation pathway that amplifies its indirect influence on health behavior.

### 3.6 Heterogeneity analysis

The above analyses were based on overall sample, but the middle-aged (45–59 years) and older adult (≥60 years) groups differ significantly in digital literacy, employment status, and physical health. Therefore, our study performed heterogeneity analysis by age group. Results are presented in Supplementary Table S3. After controlling for all covariates, the direct effect of digital literacy (DL) on health behavior was not statistically significant in either age group (45–59 years: *β* = 0.033, *p* > 0.05; ≥60 years: *β* = 0.188, *p* > 0.05). This finding is consistent with the overall conclusion that DL primarily influences health behavior through indirect paths. The mediating variables’ effects on health behavior demonstrated significant age-based heterogeneity: Proactive health awareness showed a significant positive influence on health behavior in the middle-aged group (*β* = 0.923, *p* < 0.01), but this effect was non-significant in the older adult group (*β* = 0.561, *p* > 0.05). Similarly, social capital exhibited a highly significant positive effect on health behavior for the middle-aged (*β* = 0.090, *p* < 0.001), but also lacked statistical significance for the older adults (*β* = 0.028, *p* > 0.05). In summary, the mediation paths for health behavior were primarily and significantly confined to the middle-aged group (45–59 years); statistical support for these mediation effects was insufficient among older adults (≥60 years).

## 4 Discussion

Health behaviors are essential for preserving functional capacity, improving quality of life, and reducing healthcare burdens among older adults (39). In the era of rapid digitization, older adults with higher levels of digital literacy are better equipped to retrieve credible health information, access telemedicine and health management applications, and thereby enhance their self-management capacity and psychological well-being (40). Drawing on data from CGSS 2021, this study found that digital literacy did not exert a significant direct effect on health behaviors among middle-aged and older adults. Instead, its influence was transmitted indirectly through “proactive health awareness” and “social capital,” with proactive health awareness serving as the dominant mediating mechanism (accounting for 34.98% of the total effect). A sequential pathway of “proactive health awareness → social capital → health behaviors” was also observed. These findings suggest that digital skills or access alone are insufficient to induce behavioral change; improvements in cognitive/attitudinal dimensions and the strengthening of social support networks are also indispensable (39).

The hypothesis (H1) that digital literacy directly promotes health behaviors was not supported in this study. One possible explanation is that the measurement of digital literacy was primarily focused on “access and frequency of use,” while higher-order competencies such as information comprehension and evaluative capacity (e.g., eHealth literacy) were not captured. These competencies are considered critical bridges for behavioral change (27). In addition, the adoption of health behaviors among middle-aged and older adults may be constrained by physical conditions, economic resources, and time costs. Consequently, improvements in digital skills alone may not be sufficient to overcome these “thresholds” to behavioral transformation, which may account for the lack of a direct effect observed in this study. Digital literacy may enhance individuals’ technical capacity to access information, but without corresponding improvements in self-efficacy, motivation, and health-related beliefs, such capacity alone is insufficient to induce sustained health behavior change.

The findings provided support for hypotheses H2 and H3. Proactive health awareness emerged as a core mediating mechanism in the relationship between digital literacy and health behaviors. This is consistent with the logic of the Health Belief Model, which posits that enhanced risk perception, greater self-efficacy, and stronger motivation to seek health information can translate into healthier behavioral practices (41). At the same time, social capital also acted as a significant secondary mediator. Through the expansion of social networks, increased opportunities for information exchange, and mutual support, social capital facilitated the adoption of health behaviors. This aligns with previous studies demonstrating that social participation and social support contribute to healthier lifestyles and improved health outcomes (42).

The chain mediation effect proposed in H4 was also empirically supported. Specifically, digital literacy promoted proactive health awareness, which in turn fostered the accumulation of social capital, ultimately leading to improvements in health behaviors. This suggests the existence of a compounded “psychological–social–behavioral” pathway. Enhanced health cognition appeared to activate stronger social interaction and participation, while social norms, role models, and reciprocal support within these networks reinforced and sustained health behavior. This finding enriches the theoretical explanation of how digital literacy influences health behaviors and resonates with recent studies emphasizing the role of internet use in improving older adults’ health cognition and behavioral engagement (43).

The heterogeneity analysis revealed a pronounced age-stratified pattern in the mediating role of digital literacy—through proactive health awareness and social capital—on health behaviors. The mediating effects are statistically significant only among middle-aged adults aged 45–59, whereas no significant effects were observed in the group aged 60 and above. Several factors may account for this discrepancy. First, compared with older adults, middle-aged adults generally possess stronger learning capabilities and greater cognitive flexibility (44). They are more adept at acquiring digital skills and more capable of effectively using the internet to obtain health-related information, thereby translating digital literacy into heightened health awareness and more active social participation, which ultimately promote healthier behaviors. In contrast, older adults face greater barriers to learning and declines in information-processing speed, both of which constrain their ability to convert digital competencies into effective behavioral resources. Second, differences in psychological acceptance and willingness to use digital tools may also play a role. Relative to the middle-aged adults, older adults tend to hold more conservative attitudes toward digital technologies and exhibit weaker motivation or willingness to use digital health resources, which in turn attenuates the psychological activation and social reinforcement mechanisms proposed in the model. Prior studies have similarly indicated that younger older adults usually hold a more open attitude towards new technologies (45), and this positive and open attitude enhances their capacity to benefit from digital health resources.

The findings of this study provide practical policy implications for promoting healthy aging in the digital era. First, given the crucial mediating role of proactive health awareness, policymakers should integrate structured digital health education into routine community health services, such as offering step-by-step training on using health applications, regular workshops on identifying reliable online health information, and behavior-oriented guidance for chronic disease self-management. These measures can directly strengthen health motivation and risk perception and facilitate the translation of digital literacy into everyday health practices. Second, to promote the development of social capital, community and grassroots organizations should establish stable peer-support groups, organize health-themed digital learning circles, and embed online interaction into existing community activities, thereby creating sustained channels for information exchange and mutual support among middle-aged and older adults. Finally, the observed age heterogeneity calls for age-stratified intervention strategies. For middle-aged adults with stronger learning capacity and cognitive flexibility, more advanced and interactive digital skill training can be provided to encourage deeper use of digital health services. In contrast, older adults should be offered simplified training with repeated demonstrations, on-site assistance, and follow-up support through community workers or volunteers, in order to gradually build confidence, reduce the gray digital divide, and ensure that digital skills are effectively translated into long-term health benefits (16).

There are some limitations worth noting. First, the use of cross-sectional data restricts causal inference regarding the long-term mechanisms. Second, both digital literacy and health behaviors were measured primarily through self-reported indicators, raising concerns about subjectivity and potential reporting bias, while certain dimensions (e.g., evaluative capacity, quality of application) were not fully captured. Future research should therefore: (1) employ longitudinal and experimental or quasi-experimental designs to establish causality; (2) develop and adopt more refined instruments for measuring digital literacy and objective behavioral outcomes (e.g., data from wearable devices, medical records); and (3) investigate how contextual factors, such as policy environments, technological accessibility, and cultural differences, moderate the digital literacy–health behavior link (e.g., disparities across urban–rural settings or community resource environments).

## 5 Conclusion

This study demonstrates that digital literacy exerts a significant indirect effect on health behaviors among middle-aged and older adults, with proactive health awareness serving as the principal mediating mechanism, supplemented by the accumulation of social capital. Direct improvements in digital skills alone are insufficient to guarantee behavioral change; instead, effective translation of “digitization into health improvement” requires the integration of digital skills training, health information literacy, and social support network development within both policy and community practices. To advance the goals of “Healthy China 2030” and broader digital inclusion, it is recommended to adopt an integrated intervention strategy of “technology empowerment - cognitive cultivation - social support,” and in subsequent research, these paths should be further tested for their robustness and generalization through longitudinal and experimental designs.

## Data Availability

Publicly available datasets were analyzed in this study. This data can be found at: http://cgss.ruc.edu.cn/.
