# Supplementary material for: The impact of digital literacy on health behaviors among middle-aged and older adults: the mediating roles of proactive health awareness and social capital
Source: Front Public Health. 2026 Feb 12;14:1735211. doi: 10.3389/fpubh.2026.1735211 (PMC12935962; doi:10.3389/fpubh.2026.1735211)
Supplement: Supplementary file 1 [file Table_1.DOCX]

Table 1 Digital literacy

| **Dimension** | **Question** | **Response Coding** |
| --- | --- | --- |
| Digital acquisition literacy | Do you have a mobile phone for your personal use? | Yes=1,No=0 |
| Digital access literacy | Have you accessed the internet in the past six months, including through a computer, mobile phone, or smart wearable device? | Yes=1,No=0 |
| Digital application literacy | In the past year, how often did you use the internet (including mobile internet access)? | Never=1,Rarely=2,Sometimes=3,Often =4,Very frequently=5 |
| Digital awareness literacy | Is the Internet (including mobile Internet access) your main source of information? | Yes=1,No=0 |

Table 2 Descriptive statistics of variables

| **Variable** | **Description** | **Mean** | **S. D.^1^** |
| --- | --- | --- | --- |
| **Dependent variable** |  |  |  |
| Engaged in physical exercise | Never = 1,Once a month or less = 2,Several times a month = 3,Several times a week = 4,Every day = 5 | 2.84 | 1.71 |
| Engaged in healthy diet | Never = 1,Once a month or less = 2,Several times a month = 3, Several times a week = 4,Every day = 5 | 4.59 | 0.82 |
| Health Behavior | Addition of the two above indicators | 7.43 | 2.01 |
| **Independent variable** |  |  |  |
| Digital Literacy | Comprehensive indicator | 0.49 | 0.42 |
| **Mediating variables** |  |  |  |
| Proactive Health Awareness | Comprehensive indicator | 0.19 | 0.25 |
| Engage in social or leisure activities with neighbors | Never = 1, Once a year or less = 2, Several times a year = 3, Once a month = 4, Several times a month = 5, Once or twice a week = 6, Every day = 7 | 3.83 | 2.32 |
| Engage in social or leisure activities with friends | Never = 1, Once a year or less = 2, Several times a year = 3, Once a month = 4, Several times a month = 5, Once or twice a week = 6, Every day = 7 | 3.44 | 2.03 |
| Social Capital | Addition of the two above indicators | 7.27 | 3.77 |
| **Covariates** |  |  |  |
| Gender | Male=0, Female=1 | 0.55 | 0.50 |
| Age(years) | 45 - 92 years old | 61.52 | 10.21 |
| Ethnicity | Hans=0, Minority=1 | 0.06 | 0.24 |
| Education | Illiteracy=1, Primary school=2, Junior school=3, High school and above=4 | 2.69 | 1.01 |
| Hukou | Agriculture=0, Non-agriculture=1 | 0.40 | 0.49 |
| Marital Status | Single=0, Married=1 | 0.80 | 0.40 |
| Individual Annual Income(yuan) | Less than 10000=1, 10000-29999=2, 30000 and above=3 | 2.00 | 0.88 |
| Health Status | Very poor=1, poor=2, Fair=3, Healthy=4, Very healthy=5 | 3.24 | 1.08 |

1. S.D. stands for Standard deviation

Table 3 Pearson correlation coefficients between key study variables (N=1,458).

| Variables | Health Behavior | Digital Literacy | Proactive Health Awareness | Social Capital |
| --- | --- | --- | --- | --- |
| Health Behavior | 1.000 |  |  |  |
| Digital Literacy | 0.125^***^ | 1.000 |  |  |
| Proactive Health Awareness | 0.173^***^ | 0.592^***^ | 1.000 |  |
| Social Capital | 0.127^***^ | 0.119^***^ | 0.126^***^ | 1.000 |
| *** p<0.001, ** p<0.01, * p<0.05 | | | | |

Table 4 Impact of digital literacy on the health behavior of middle-aged and older adults

| **Variables** | **Proactive Health Awareness** | |  | **Social Capital** | |  | **Health Behavior** | |
| --- | --- | --- | --- | --- | --- | --- | --- | --- |
|  | **Model 1**  **(Unadjusted)** | **Model 2**  **(Adjusted)** |  | **Model 3**  **(Unadjusted)** | **Model 4**  **(Adjusted)** |  | **Model 5**  **(Unadjusted)** | **Model 6**  **(Adjusted)** |
| **Digital Literacy** | 0.354*** | 0.296*** |  | 0.619* | 0.772* |  | 0.133 | 0.073 |
|  | (0.012) | (0.016) |  | (0.291) | (0.333) |  | (0.157) | (0.174) |
| **Proactive Health Awareness** | - | - |  | 1.291** | 1.445** |  | 1.155*** | 0.777** |
|  |  |  |  | (0.481) | (0.495) |  | (0.250) | (0.261) |
| **Social Capital** | - | - |  | - | - |  | 0.056*** | 0.056*** |
|  |  |  |  |  |  |  | (0.014) | (0.014) |
| **Gender(Male ^Ref^)** | | |  |  |  |  |  |  |
| Female |  | 0.008 |  |  | 0.012 |  |  | 0.028 |
|  |  | (0.011) |  |  | (0.207) |  |  | (0.108) |
| **Age/years** |  | 0.001 |  |  | -1.086** |  |  | 0.007 |
|  |  | (0.001) |  |  | (0.370) |  |  | (0.006) |
| **Ethnicity(Hans ^Ref^)** | | |  |  |  |  |  |  |
| Minority |  | 0.009 |  |  | 0.013 |  |  | 0.050 |
|  |  | (0.018) |  |  | (0.326) |  |  | (0.204) |
| **Education(Illiteracy ^Ref^)** | | |  |  | 0.104 |  |  |  |
| Primary school |  | 0.018 |  |  | (0.339) |  |  | 0.050 |
|  |  | (0.012) |  |  | -0.266 |  |  | (0.204) |
| Junior school |  | 0.057*** |  |  | (0.388) |  |  | 0.050 |
|  |  | (0.014) |  |  |  |  |  | (0.204) |
| High school and above |  | 0.105*** |  |  | -0.578* |  |  | 0.050 |
|  |  | (0.018) |  |  | (0.256) |  |  | (0.204) |
| **Hukou(Agriculture ^Ref^)** | | |  |  |  |  |  |  |
| Non-agriculture |  | 0.031* |  |  | -0.165 |  |  | 0.247 |
|  |  | (0.014) |  |  | (0.263) |  |  | (0.128) |
| **Marital Status(Single ^Ref^)** | | |  |  |  |  |  |  |
| Married |  | 0.012 |  |  | -0.028 |  |  | 0.277* |
|  |  | (0.012) |  |  | (0.284) |  |  | (0.133) |
| **Individual Annual Income/yuan(Less than 10000 ^Ref^)** | | | | | 0.261 |  |  |  |
| 10000-29999 |  | -0.004 |  |  | (0.286) |  |  | -0.008 |
|  |  | (0.013) |  |  |  |  |  | (0.145) |
| 30000 and above |  | 0.027 |  |  | 0.663 |  |  | -0.107 |
|  |  | (0.015) |  |  | (0.455) |  |  | (0.152) |
| **Health Status( Very poor ^Ref^)** | |  |  |  | 0.863* |  |  |  |
| Poor |  | -0.003 |  |  | (0.431) |  |  | 0.441 |
|  |  | (0.018) |  |  | 0.927* |  |  | (0.252) |
| Fair |  | 0.012 |  |  | (0.433) |  |  | 0.879*** |
|  |  | (0.018) |  |  | 1.028* |  |  | (0.239) |
| Healthy |  | 0.022 |  |  | (0.495) |  |  | 0.876*** |
|  |  | (0.019) |  |  | 1,458 |  |  | (0.241) |
| Very healthy |  | 0.024 |  |  | 0.034 |  |  | 1.029*** |
|  |  | (0.023) |  |  | 0.772* |  |  | (0.269) |
| **Observations** | 1,458 | 1,458 |  | 1,458 | (0.333) |  | 1,458 | 1,458 |
| **R-squared** | 0.350 | 0.395 |  | 0.019 | 1.445** |  | 0.042 | 0.078 |
| Robust standard errors in parentheses；*** p<0.001, ** p<0.01, * p<0.05; All variance inflation factors (VIF) were below 5, indicating no serious multicollinearity. | | | | | | | | |

**SUPPLEMENTARY**

Supplementary Table 1 Robustness analysis

| **Variables** | **OLR** | |  | **Oprobit** | |
| --- | --- | --- | --- | --- | --- |
|  | **Model 7**  **(Unadjusted)** | **Model 8**  **(Adjusted)** |  | **Model 9**  **(Unadjusted)** | **Model 10**  **(Adjusted)** |
| **Digital Literacy** | 0.134 | 0.099 |  | 0.054 | 0.027 |
|  | (0.142) | (0.160) |  | (0.084) | (0.094) |
| **Proactive Health Awareness** | 1.025*** | 0.688** |  | 0.634*** | 0.439** |
|  | (0.230) | (0.247) |  | (0.138) | (0.146) |
| **Social Capital** | 0.051*** | 0.053*** |  | 0.031*** | 0.031*** |
|  | (0.012) | (0.013) |  | (0.007) | (0.007) |
| **Gender(Male ^Ref^)** |  |  |  |  |  |
| Female |  | -0.002 |  |  | 0.016 |
|  |  | (0.101) |  |  | (0.059) |
| **Age/years** |  | 0.008 |  |  | 0.004 |
|  |  | (0.006) |  |  | (0.003) |
| **Ethnicity(Hans ^Ref^)** |  |  |  |  |  |
| Minority |  | 0.079 |  |  | 0.039 |
|  |  | (0.196) |  |  | (0.113) |
| **Education(Illiteracy ^Ref^)** | |  |  |  |  |
| Primary school |  | 0.160 |  |  | 0.098 |
|  |  | (0.162) |  |  | (0.092) |
| Junior school |  | 0.252 |  |  | 0.145 |
|  |  | (0.164) |  |  | (0.094) |
| High school and above |  | 0.496** |  |  | 0.274* |
|  |  | (0.191) |  |  | (0.112) |
| **Hukou(Agriculture ^Ref^)** | |  |  |  |  |
| Non-agriculture |  | 0.211 |  |  | 0.137* |
|  |  | (0.118) |  |  | (0.069) |
| **Marital Status(Single ^Ref^)** | |  |  |  |  |
| Married |  | 0.221 |  |  | 0.158* |
|  |  | (0.123) |  |  | (0.072) |
| **Individual Annual Income/yuan(Less than 10000 ^Ref^)** | | | | |  |
| 10000-29999 |  | -0.018 |  |  | 0.019 |
|  |  | (0.133) |  |  | (0.077) |
| 30000 and above |  | -0.099 |  |  | -0.037 |
|  |  | (0.142) |  |  | (0.082) |
| **Health Status(Very poor ^Ref^)** | |  |  |  |  |
| Poor |  | 0.371 |  |  | 0.228 |
|  |  | (0.251) |  |  | (0.136) |
| Fair |  | 0.828*** |  |  | 0.469*** |
|  |  | (0.239) |  |  | (0.130) |
| Healthy |  | 0.840*** |  |  | 0.485*** |
|  |  | (0.239) |  |  | (0.131) |
| Very healthy |  | 0.977*** |  |  | 0.570*** |
|  |  | (0.265) |  |  | (0.147) |
| Robust standard errors in parentheses；*** p<0.001, ** p<0.01, * p<0.05 | | | | | |

Supplementary Table 2 Dimensional analysis

| **Variables** | **Engaged in physical exercise** | |  | **Engaged in healthy diet** | |
| --- | --- | --- | --- | --- | --- |
|  | **Model 11**  **(Unadjusted)** | **Model 12**  **(Adjusted)** |  | **Model 13**  **(Unadjusted)** | **Model 14**  **(Adjusted)** |
| **Digital Literacy** | 0.174 | 0.053 |  | -0.041 | -0.069 |
|  | (0.132) | (0.150) |  | (0.069) | (0.079) |
| **Proactive Health Awareness** | 0.877*** | 0.616** |  | 0.278** | 0.161 |
|  | (0.218) | (0.229) |  | (0.103) | (0.105) |
| **Social Capital** | 0.043*** | 0.043*** |  | 0.013* | 0.013* |
|  | (0.012) | (0.012) |  | (0.006) | (0.006) |
| **Gender(Male ^Ref^)** |  |  |  |  |  |
| Female |  | -0.125 |  |  | 0.153** |
|  |  | (0.092) |  |  | (0.047) |
| **Age/years** |  | 0.002 |  |  | 0.005* |
|  |  | (0.005) |  |  | (0.003) |
| **Ethnicity(Hans ^Ref^)** |  |  |  |  |  |
| Minority |  | -0.091 |  |  | 0.141 |
|  |  | (0.168) |  |  | (0.085) |
| **Education(Illiteracy ^Ref^)** | |  |  |  |  |
| Primary school |  | 0.169 |  |  | 0.047 |
|  |  | (0.142) |  |  | (0.078) |
| Junior school |  | 0.193 |  |  | 0.118 |
|  |  | (0.148) |  |  | (0.077) |
| High school and above |  | 0.478** |  |  | 0.083 |
|  |  | (0.172) |  |  | (0.088) |
| **Hukou(Agriculture ^Ref^)** | |  |  |  |  |
| Non-agriculture |  | 0.132 |  |  | 0.115* |
|  |  | (0.113) |  |  | (0.050) |
| **Marital Status(Single ^Ref^)** | |  |  |  |  |
| Married |  | 0.112 |  |  | 0.164** |
|  |  | (0.109) |  |  | (0.060) |
| **Individual Annual Income/yuan(Less than 10000 ^Ref^)** | | |  |  |  |
| 10000-29999 |  | -0.075 |  |  | 0.067 |
|  |  | (0.126) |  |  | (0.060) |
| 30000 and above |  | -0.138 |  |  | 0.031 |
|  |  | (0.128) |  |  | (0.063) |
| **Health Status(Very poor ^Ref^)** | |  |  |  |  |
| Poor |  | 0.115 |  |  | 0.326* |
|  |  | (0.204) |  |  | (0.131) |
| Fair |  | 0.507** |  |  | 0.372** |
|  |  | (0.196) |  |  | (0.127) |
| Healthy |  | 0.456* |  |  | 0.420*** |
|  |  | (0.198) |  |  | (0.127) |
| Very healthy |  | 0.548* |  |  | 0.481*** |
|  |  | (0.225) |  |  | (0.134) |
| **Observations** | 1,458 | 1,458 |  | 1,458 | 1,458 |
| **R-squared** | 0.037 | 0.062 |  | 0.010 | 0.053 |
| Robust standard errors in parentheses；*** p<0.001, ** p<0.01, * p<0.05 | | | | | |

Supplementary Table 3 Heterogeneity analysis

| **Variables** | **Age[45-59]** | |  | **Age≥60** | |
| --- | --- | --- | --- | --- | --- |
|  | **Model 15**  **(Unadjusted)** | **Model 16**  **(Adjusted)** |  | **Model 17**  **(Unadjusted)** | **Model 18**  **(Adjusted)** |
| **Digital Literacy** | 0.072 | 0.033 |  | 0.500 | 0.188 |
|  | (0.228) | (0.230) |  | (0.259) | (0.269) |
| **Proactive Health Awareness** | 1.309*** | 0.923** |  | 0.892* | 0.561 |
|  | (0.316) | (0.335) |  | (0.406) | (0.427) |
| **Social Capital** | 0.089*** | 0.090*** |  | 0.029 | 0.028 |
|  | (0.020) | (0.019) |  | (0.019) | (0.019) |
| **Gender(Male ^Ref^)** |  |  |  |  |  |
| Female |  | 0.154 |  |  | -0.046 |
|  |  | (0.152) |  |  | (0.157) |
| **Ethnicity(Hans ^Ref^)** |  |  |  |  |  |
| Minority |  | 0.358 |  |  | -0.228 |
|  |  | (0.284) |  |  | (0.288) |
| **Education(Illiteracy ^Ref^)** | |  |  |  |  |
| Primary school |  | 0.040 |  |  | 0.289 |
|  |  | (0.284) |  |  | (0.220) |
| Junior school |  | 0.121 |  |  | 0.385 |
|  |  | (0.280) |  |  | (0.232) |
| High school and above |  | 0.545 |  |  | 0.439 |
|  |  | (0.310) |  |  | (0.285) |
| **Hukou(Agriculture ^Ref^)** | |  |  |  |  |
| Non-agriculture |  | 0.188 |  |  | 0.232 |
|  |  | (0.165) |  |  | (0.210) |
| **Marital Status(Single ^Ref^)** | |  |  |  |  |
| Married |  | 0.184 |  |  | 0.295 |
|  |  | (0.214) |  |  | (0.169) |
| **Individual Annual Income/yuan(Less than 10000 ^Ref^)** | | |  |  |  |
| 10000-29999 |  | -0.224 |  |  | 0.312 |
|  |  | (0.198) |  |  | (0.210) |
| 30000 and above |  | -0.164 |  |  | -0.009 |
|  |  | (0.210) |  |  | (0.240) |
| **Health Status(Very poor ^Ref^)** | |  |  |  |  |
| Poor |  | 0.522 |  |  | 0.390 |
|  |  | (0.410) |  |  | (0.307) |
| Fair |  | 0.692 |  |  | 1.037*** |
|  |  | (0.378) |  |  | (0.299) |
| Healthy |  | 0.779* |  |  | 0.978** |
|  |  | (0.383) |  |  | (0.301) |
| Very healthy |  | 1.000* |  |  | 0.989** |
|  |  | (0.408) |  |  | (0.364) |
| **Observations** | 712 | 712 |  | 746 | 746 |
| **R-squared** | 0.066 | 0.096 |  | 0.034 | 0.090 |
| Robust standard errors in parentheses；*** p<0.001, ** p<0.01, * p<0.05 | | | | | |
