# Supplementary material for: The impact of digital literacy on health behaviors among middle-aged and older adults: the mediating roles of proactive health awareness and social capital
Source: Front Public Health. 2026 Feb 12;14:1735211. doi: 10.3389/fpubh.2026.1735211 (PMC12935962; doi:10.3389/fpubh.2026.1735211)
Supplement: Supplementary file 2 [file Table_2.DOCX]

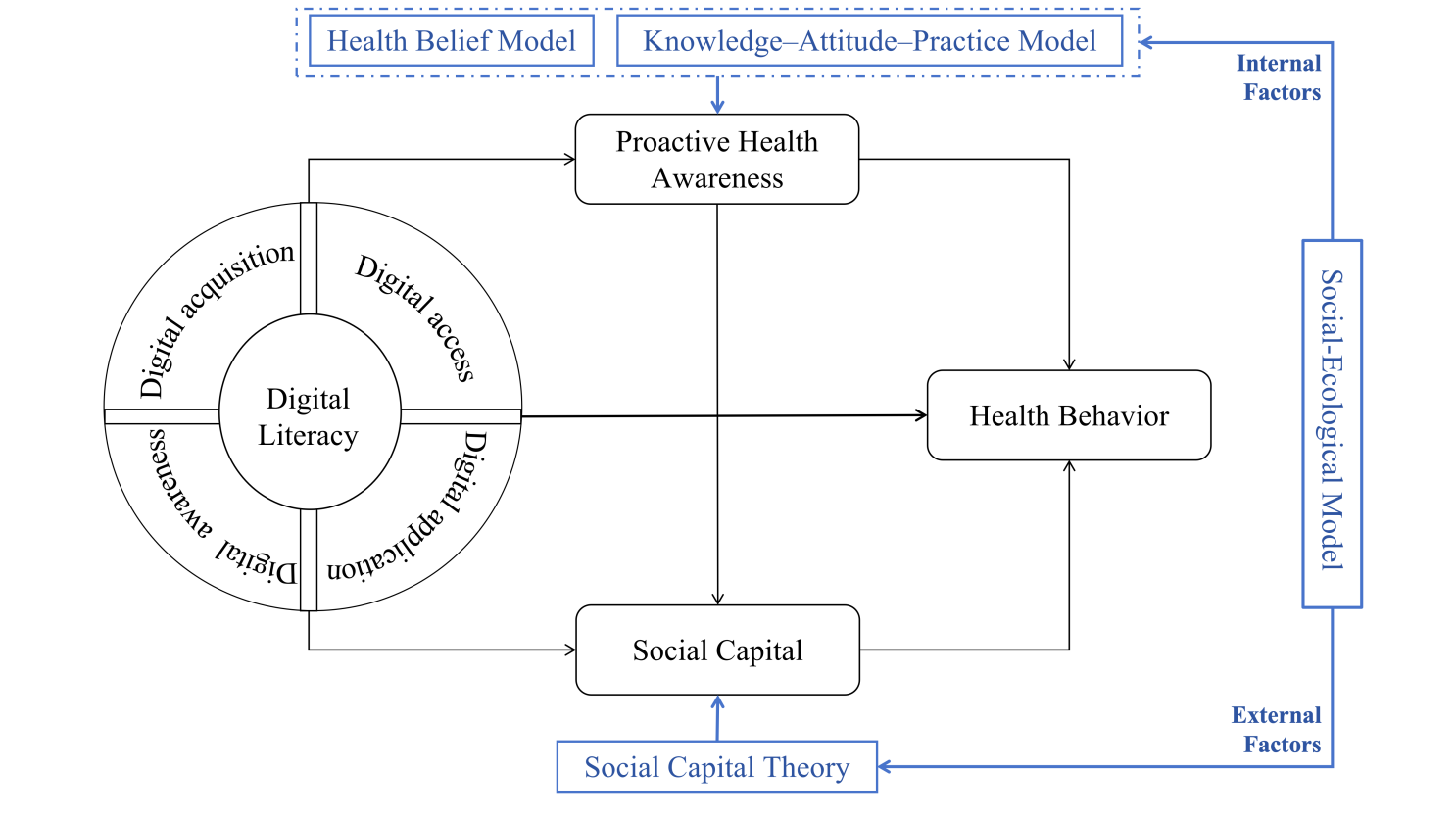
Figure 1 Theoretical analysis diagram


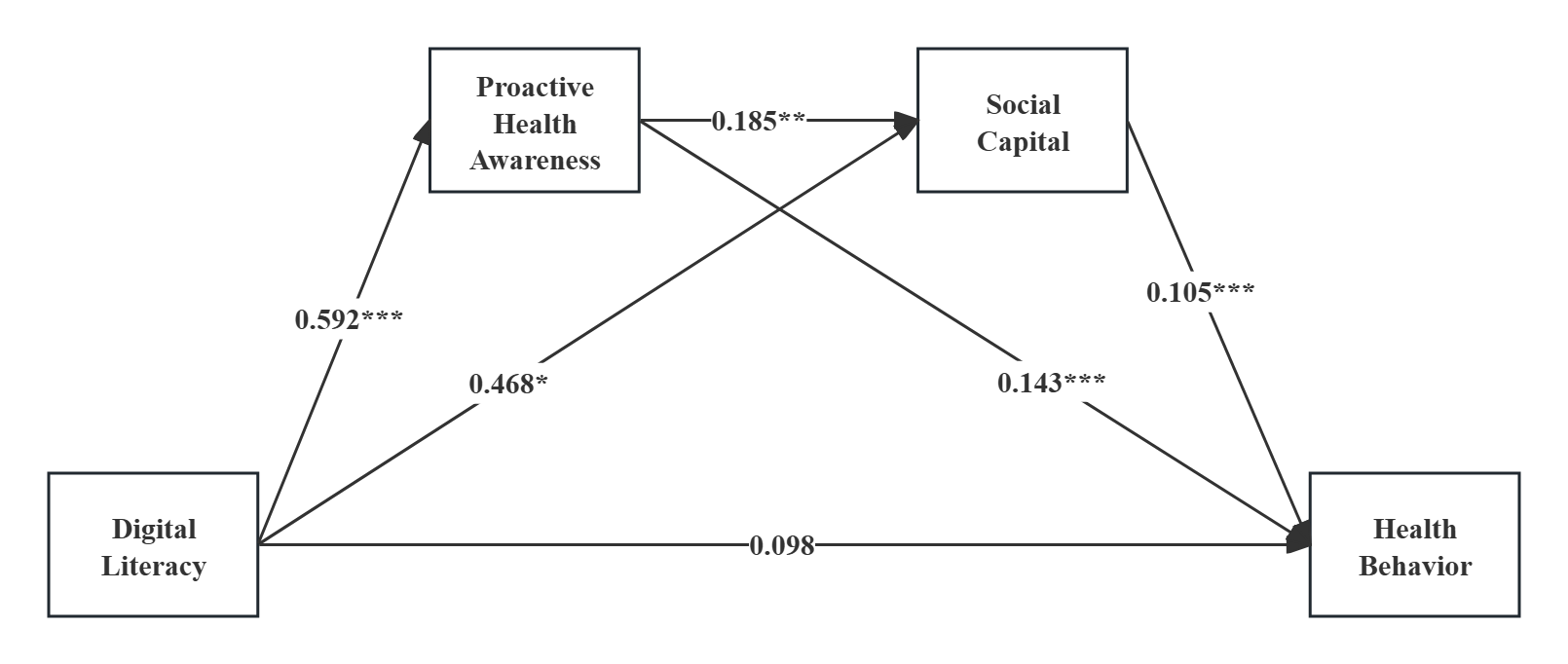


Figure 2 A multiple mediation model of the association between digital literacy and health behavior throng proactive health awareness and social capital.
Note, Standard path coefficients are shown, *** p<0.001, ** p<0.01, * p<0.05
