# Supplementary material for: The impact of digital literacy on health behaviors among middle-aged and older adults: the mediating roles of proactive health awareness and social capital
Source: Front Public Health. 2026 Feb 12;14:1735211. doi: 10.3389/fpubh.2026.1735211 (PMC12935962; doi:10.3389/fpubh.2026.1735211)
Supplement: Supplementary file 3 [file Table_3.DOCX]

**SUPPLEMENTARY**

Supplementary Table 1 Robustness analysis

| **Variables** | **OLR** | |  | **Oprobit** | |
| --- | --- | --- | --- | --- | --- |
|  | **Model 7**  **(Unadjusted)** | **Model 8**  **(Adjusted)** |  | **Model 9**  **(Unadjusted)** | **Model 10**  **(Adjusted)** |
| **Digital Literacy** | 0.134 | 0.099 |  | 0.054 | 0.027 |
|  | (0.142) | (0.160) |  | (0.084) | (0.094) |
| **Proactive Health Awareness** | 1.025*** | 0.688** |  | 0.634*** | 0.439** |
|  | (0.230) | (0.247) |  | (0.138) | (0.146) |
| **Social Capital** | 0.051*** | 0.053*** |  | 0.031*** | 0.031*** |
|  | (0.012) | (0.013) |  | (0.007) | (0.007) |
| **Gender(Male ^Ref^)** |  |  |  |  |  |
| Female |  | -0.002 |  |  | 0.016 |
|  |  | (0.101) |  |  | (0.059) |
| **Age/years** |  | 0.008 |  |  | 0.004 |
|  |  | (0.006) |  |  | (0.003) |
| **Ethnicity(Hans ^Ref^)** |  |  |  |  |  |
| Minority |  | 0.079 |  |  | 0.039 |
|  |  | (0.196) |  |  | (0.113) |
| **Education(Illiteracy ^Ref^)** | |  |  |  |  |
| Primary school |  | 0.160 |  |  | 0.098 |
|  |  | (0.162) |  |  | (0.092) |
| Junior school |  | 0.252 |  |  | 0.145 |
|  |  | (0.164) |  |  | (0.094) |
| High school and above |  | 0.496** |  |  | 0.274* |
|  |  | (0.191) |  |  | (0.112) |
| **Hukou(Agriculture ^Ref^)** | |  |  |  |  |
| Non-agriculture |  | 0.211 |  |  | 0.137* |
|  |  | (0.118) |  |  | (0.069) |
| **Marital Status(Single ^Ref^)** | |  |  |  |  |
| Married |  | 0.221 |  |  | 0.158* |
|  |  | (0.123) |  |  | (0.072) |
| **Individual Annual Income/yuan(Less than 10000 ^Ref^)** | | | | |  |
| 10000-29999 |  | -0.018 |  |  | 0.019 |
|  |  | (0.133) |  |  | (0.077) |
| 30000 and above |  | -0.099 |  |  | -0.037 |
|  |  | (0.142) |  |  | (0.082) |
| **Health Status(Very poor ^Ref^)** | |  |  |  |  |
| Poor |  | 0.371 |  |  | 0.228 |
|  |  | (0.251) |  |  | (0.136) |
| Fair |  | 0.828*** |  |  | 0.469*** |
|  |  | (0.239) |  |  | (0.130) |
| Healthy |  | 0.840*** |  |  | 0.485*** |
|  |  | (0.239) |  |  | (0.131) |
| Very healthy |  | 0.977*** |  |  | 0.570*** |
|  |  | (0.265) |  |  | (0.147) |
| Robust standard errors in parentheses；*** p<0.001, ** p<0.01, * p<0.05 | | | | | |

Supplementary Table 2 Dimensional analysis

| **Variables** | **Engaged in physical exercise** | |  | **Engaged in healthy diet** | |
| --- | --- | --- | --- | --- | --- |
|  | **Model 11**  **(Unadjusted)** | **Model 12**  **(Adjusted)** |  | **Model 13**  **(Unadjusted)** | **Model 14**  **(Adjusted)** |
| **Digital Literacy** | 0.174 | 0.053 |  | -0.041 | -0.069 |
|  | (0.132) | (0.150) |  | (0.069) | (0.079) |
| **Proactive Health Awareness** | 0.877*** | 0.616** |  | 0.278** | 0.161 |
|  | (0.218) | (0.229) |  | (0.103) | (0.105) |
| **Social Capital** | 0.043*** | 0.043*** |  | 0.013* | 0.013* |
|  | (0.012) | (0.012) |  | (0.006) | (0.006) |
| **Gender(Male ^Ref^)** |  |  |  |  |  |
| Female |  | -0.125 |  |  | 0.153** |
|  |  | (0.092) |  |  | (0.047) |
| **Age/years** |  | 0.002 |  |  | 0.005* |
|  |  | (0.005) |  |  | (0.003) |
| **Ethnicity(Hans ^Ref^)** |  |  |  |  |  |
| Minority |  | -0.091 |  |  | 0.141 |
|  |  | (0.168) |  |  | (0.085) |
| **Education(Illiteracy ^Ref^)** | |  |  |  |  |
| Primary school |  | 0.169 |  |  | 0.047 |
|  |  | (0.142) |  |  | (0.078) |
| Junior school |  | 0.193 |  |  | 0.118 |
|  |  | (0.148) |  |  | (0.077) |
| High school and above |  | 0.478** |  |  | 0.083 |
|  |  | (0.172) |  |  | (0.088) |
| **Hukou(Agriculture ^Ref^)** | |  |  |  |  |
| Non-agriculture |  | 0.132 |  |  | 0.115* |
|  |  | (0.113) |  |  | (0.050) |
| **Marital Status(Single ^Ref^)** | |  |  |  |  |
| Married |  | 0.112 |  |  | 0.164** |
|  |  | (0.109) |  |  | (0.060) |
| **Individual Annual Income/yuan(Less than 10000 ^Ref^)** | | |  |  |  |
| 10000-29999 |  | -0.075 |  |  | 0.067 |
|  |  | (0.126) |  |  | (0.060) |
| 30000 and above |  | -0.138 |  |  | 0.031 |
|  |  | (0.128) |  |  | (0.063) |
| **Health Status(Very poor ^Ref^)** | |  |  |  |  |
| Poor |  | 0.115 |  |  | 0.326* |
|  |  | (0.204) |  |  | (0.131) |
| Fair |  | 0.507** |  |  | 0.372** |
|  |  | (0.196) |  |  | (0.127) |
| Healthy |  | 0.456* |  |  | 0.420*** |
|  |  | (0.198) |  |  | (0.127) |
| Very healthy |  | 0.548* |  |  | 0.481*** |
|  |  | (0.225) |  |  | (0.134) |
| **Observations** | 1,458 | 1,458 |  | 1,458 | 1,458 |
| **R-squared** | 0.037 | 0.062 |  | 0.010 | 0.053 |
| Robust standard errors in parentheses；*** p<0.001, ** p<0.01, * p<0.05 | | | | | |

Supplementary Table 3 Heterogeneity analysis

| **Variables** | **Age[45-59]** | |  | **Age≥60** | |
| --- | --- | --- | --- | --- | --- |
|  | **Model 15**  **(Unadjusted)** | **Model 16**  **(Adjusted)** |  | **Model 17**  **(Unadjusted)** | **Model 18**  **(Adjusted)** |
| **Digital Literacy** | 0.072 | 0.033 |  | 0.500 | 0.188 |
|  | (0.228) | (0.230) |  | (0.259) | (0.269) |
| **Proactive Health Awareness** | 1.309*** | 0.923** |  | 0.892* | 0.561 |
|  | (0.316) | (0.335) |  | (0.406) | (0.427) |
| **Social Capital** | 0.089*** | 0.090*** |  | 0.029 | 0.028 |
|  | (0.020) | (0.019) |  | (0.019) | (0.019) |
| **Gender(Male ^Ref^)** |  |  |  |  |  |
| Female |  | 0.154 |  |  | -0.046 |
|  |  | (0.152) |  |  | (0.157) |
| **Ethnicity(Hans ^Ref^)** |  |  |  |  |  |
| Minority |  | 0.358 |  |  | -0.228 |
|  |  | (0.284) |  |  | (0.288) |
| **Education(Illiteracy ^Ref^)** | |  |  |  |  |
| Primary school |  | 0.040 |  |  | 0.289 |
|  |  | (0.284) |  |  | (0.220) |
| Junior school |  | 0.121 |  |  | 0.385 |
|  |  | (0.280) |  |  | (0.232) |
| High school and above |  | 0.545 |  |  | 0.439 |
|  |  | (0.310) |  |  | (0.285) |
| **Hukou(Agriculture ^Ref^)** | |  |  |  |  |
| Non-agriculture |  | 0.188 |  |  | 0.232 |
|  |  | (0.165) |  |  | (0.210) |
| **Marital Status(Single ^Ref^)** | |  |  |  |  |
| Married |  | 0.184 |  |  | 0.295 |
|  |  | (0.214) |  |  | (0.169) |
| **Individual Annual Income/yuan(Less than 10000 ^Ref^)** | | |  |  |  |
| 10000-29999 |  | -0.224 |  |  | 0.312 |
|  |  | (0.198) |  |  | (0.210) |
| 30000 and above |  | -0.164 |  |  | -0.009 |
|  |  | (0.210) |  |  | (0.240) |
| **Health Status(Very poor ^Ref^)** | |  |  |  |  |
| Poor |  | 0.522 |  |  | 0.390 |
|  |  | (0.410) |  |  | (0.307) |
| Fair |  | 0.692 |  |  | 1.037*** |
|  |  | (0.378) |  |  | (0.299) |
| Healthy |  | 0.779* |  |  | 0.978** |
|  |  | (0.383) |  |  | (0.301) |
| Very healthy |  | 1.000* |  |  | 0.989** |
|  |  | (0.408) |  |  | (0.364) |
| **Observations** | 712 | 712 |  | 746 | 746 |
| **R-squared** | 0.066 | 0.096 |  | 0.034 | 0.090 |
| Robust standard errors in parentheses；*** p<0.001, ** p<0.01, * p<0.05 | | | | | |
